# The role of mouse models in colorectal cancer research—The need and the importance of the orthotopic models

**DOI:** 10.1002/ame2.12102

**Published:** 2020-03-11

**Authors:** Rui C. Oliveira, Ana Margarida Abrantes, José Guilherme Tralhão, Maria Filomena Botelho

**Affiliations:** ^1^ Biophysics Unit Faculty of Medicine University of Coimbra Coimbra Portugal; ^2^ Pathology Department University Hospital (CHUC) Coimbra Portugal; ^3^ Centre of Investigation on Environment, Genetics and Oncobiology (CIMAGO) Coimbra Portugal; ^4^ Surgery A Department Faculty of Medicine University Hospital (CHUC) Coimbra Portugal

**Keywords:** colorectal cancer, mouse model, orthotopic model

## Abstract

Colorectal cancer is a worldwide health burden, with high incidence and mortality, especially in the advanced stages of the disease. Preclinical models are very important and valuable to discover and validate early and specific biomarkers as well as new therapeutic targets. In order to accomplish that, the animal models must replicate the clinical evolution of the disease in all of its phases. In this article, we review the existent mouse models, with their strengths and weaknesses in the replication of human cancer disease progression, with major focus on orthotopic models.

## 1 INTRODUCTION

Colorectal cancer (CRC) is a major medical concern, being the third most common cancer type and the fourth most common cause of cancer‐related death, accounting for 9% of the total cancer incidence.^1^ In spite of the progress in clinical and biological knowledge, CRC remains a main public health issue,^2^ and despite the rapid development of treatments in the last years, the mortality rate related to this type of cancer remains high,^3^ especially in the advanced stages of disease.^4^ About half of the patients with CRC will develop liver metastases, and from that subpopulation only 25% are eligible for surgery with curative intent.^5^

The staging of CRC is the most important prognostic factor for survival, and when patients are in an advanced stage (with the development of metastasis), the prognosis is extremely poor and survival is estimated in months.^6^ Surgery and chemotherapy have been valuable allies in the treatment of CRC, and are able to treat 75% of patients, but more than 30% of these patients develop new neoplastic lesions, and 10% evolve to second malignancy.^7^

Nowadays it is clear that CRC is a heterogeneous disease that is caused by changes in different complex pathogenic pathways^8^ and molecular changes in a multistep carcinogenesis cascade that differ from tumor to tumor and reveal a wide range of clinical behaviors.^9^ At a molecular level, CRC is the tip of the iceberg of an intricate and ample array of gene alterations, affecting supramolecular processes.^2^

Therefore, it is understandable that CRC is a composite and multistep process, and when it is put together with recent discoveries in the CRC carcinogenesis, like the influence of the tumor microenvironment in primary and secondary tumor development and possible applications in CRC treatment,^10^, ^11^, ^12^, ^13^, ^14^, ^15^ together with the existence and influence of cancer stem cells in tumor progression, aggressiveness, and resistance to therapeutics,^6^, ^16^ new data, possibilities, and major dilemmas in CRC carcinogenesis must be taken into consideration.

So, the expansion of new strategies of screening that allow early and higher rates of CRC detection and development and creation of new preclinical models for the study of the CRC carcinogenesis process with the discovery of sensitive and specific biomarkers, not only for initial detection, but also to identify patients who will have disease recurrence and patients who will probably progress despite adjuvant therapy are critical and essentials steps in managing CRC.^17^, ^18^

## 2 WHY THE ANIMAL MODEL?

Despite differences in animals, their genomes are built on DNA, the chemical basis of life. By comparing the human genome with animal genome, it is possible to better understand the structure and function of human genes, and apply that knowledge to study human diseases in order to develop new strategies and mechanisms to prevent, detect, and treat CRC. In this context, several animals had their genome sequenced, such as the mouse (*Mus musculus*), the fruit fly (*Drosophila melanogaster*), and the malaria‐carrying mosquito (*Anopheles gambiae*), among others.^19^

Of all those animals, the mouse is the most used animal model in the study of carcinogenesis, with its use as the main model in biomedical research dating back to the beginning of the human civilization, when humans recorded coat‐color mutations for millennia. In 1700, in China and Japan, mice were domesticated as pets, and then imported by Europeans who bred them with their local varieties, creating hybrid progenitors of modern laboratory mice.^20^ Application of Mendel's law of inheritance to mice in an analogue way to the peas gave birth to new theories of inheritance, and with DNA recombinant technologies/DNA sequence‐based polymorphisms, new models were created.^20^

Although yeasts and flies reveled themselves as excellent models for studying cell cycle, mice are better models for studying the immune, endocrine, nervous, and other physiological systems, because their genetic and physiology are closer to those of humans. Like humans, mice have the ability to develop several diseases, such as cancer. In recent times, the use of innovative genetic technologies enabled the production of transgenic mice, with gene insertion in the germinal line, and even more advanced changes with “knock out” and “knock in” genes (eg, p53 knockout with disabled TP53 tumor suppressor gene), allied with state of the art reproductive technologies, provided the necessary tools for a deep study of carcinogenesis mechanisms in mice.^21^

An ideal animal CRC model should allow the development of local tumors, it should replicate all stages of CRC evolution, permit the assessment of the disease progression with radiology and endoscopic methods, (it should allow us to) understand the toxicities related with therapeutic procedures, and be largely adjustable to laboratories without surgical knowledge, enabling its replication.^5^, ^22^ However, the development of animal models is an arduous work, depending majorly on the strain of animals and cancer cells that are used.^23^

Therefore, mice are very valuable in cancer investigation and many transgenic mice were created in order to provide models to study the development and behavior of different types of tumors (gastric, pancreatic, bone, colon, etc). In this article, mouse CRC models will be discussed in detail, with special focus on orthotopic models.

## 3 WHAT TYPE OF CRC MODELS EXIST?

### 3.1 Sporadic CRC models/chemical‐induced models

Several models of mouse CRC have been developed along the time. Perhaps the first model developed was one that demonstrated the relation between intestinal tumor genesis and the ingestion of polycyclic aromatic hydrocarbon.^24^ At that time, the relation between CRC development and the ingestion of some types of food was under keen study, creating sporadic CRC models; more models were developed according to this idea: ingestion of radioactive yttrium^25^ of 4‐aminodephenyl and 3,2‐dimethyl‐4‐aminodiphenyl^26^ and 1,2‐dimethylhydrazine azoxymethane.^27^, ^28^, ^29^ In time, other carcinogens that induced colon tumors were discovered, such as heterocyclic and aromatic amines, and alkylnitrosamide compounds. A recent and one of the most used sporadic CRC models promotes CRC by chronic use of dextran sodium sulfate, recreating the conditions of an inflammatory bowel disease.^30^, ^31^

These models, despite their utility as pioneers in the first step of tumor carcinogenesis, are extremely limited, because they have low tumor development—only a fraction of the mice that experienced those conditions developed tumors, and when these develop, they reveal a widely variability in location, diffusion, and differentiation.^32^

The relation between exposure to carcinogens and tumor development is well‐know, and the above described models are still used, but it was necessary to create new and more “profitable” ones.

In order to overcome the low tumor development rate, a simple strategy was formulated. Instead of exposing the animals to carcinogenic elements, why not expose them directly to cancer cells? Why not study the behavior of cancer cells? And why not expose them to carcinogenic elements with in situ application? This train of thought gave birth to new type of cancer models which require surgical skill and the application of cancer elements (tissue/cells) directly in the animal as well as in vitro studies.

Many models were then created, developed, and improved by the discoveries and comprehension of CRC pathways and progression.

### 3.2 Cell culture models

One of the main and most used models is the human cancer cell lines model. Cell lines harvested from tumor tissue from patients with CRC are used to model the disease, and there are even cell lines established for this objective. Since the cells have origin in human tissue they provide high fidelity and allow various types of experiences relevant for human disease, and they are not very expensive—a major attractive quality. This model allows to mimic/to replicate tumor cells behavior in culture, providing further analysis of several aspects such as: aggregation, migration, colonies formation, responsiveness to therapeutics, and even to measure the production of intercellular messengers; it is also possible to evaluate parameters like oxidative stress, viability and apoptosis, cell cycle analysis, and determination of surface antigens.^33^, ^34^, ^35^, ^36^, ^37^

A major disadvantage of this model is the fact that it does not reproduce the tumor cell/environment interaction. The environment in culture is artificial and does not simulate the host response to tumor presence—immune response and angiogenesis.^34^ Also, tumor cells are maintained over many passages lacking intra‐tumoral heterogeneity and regularly showing few similarities, physiological and genetic, to the tumor from which they derive.^38^

Despite this drawback, the cell culture is a widely used model to study human carcinomas and a great complement to other models, but in order for the study to be accurate, the tumor must be preferentially studied in vivo.

### 3.3 Carcinogens in situ

A way to overcome the limitation of the sporadic cancer models with ingestion of carcinogens was to bypass the digestive tract, eliminating enzymatic alterations, and introduce them directly into the desired place—commonly by intrarectal exposure.^38^ This model reveals greater efficiency than sporadic CRC models, but the income on tumor formation is still far from the ideal. However, this model type had its efficiency greatly improved with the creation of genetic mouse models, but even so, in some cases, when the metastases are detected, the animals are too ill to expose them to therapeutic options.^5^

### 3.4 Peritoneum models

The injection of tumor cells into the peritoneum and peritoneal cavity was probably one of the first ideas in order to promote tumor development in mice and its study. It is still used nowadays leading to the development of tumor nodules on the peritoneum and dissemination through the peritoneal cavity.^39^

However, its biological behavior does not mimic/replicate the human CRC and other solid tumors, leaving the peritoneum models a tool for evaluating nonsolid tumors like leukemia's and to assess pharmacologic responses,^40^ since peritoneal metastatic carcinoma has worse prognosis compared with other metastasis and the treatments available did not achieve/have not achieved a good rate of effectiveness yet.^41^

### 3.5 Xenografts and orthotopic models

**FIGURE 1:**
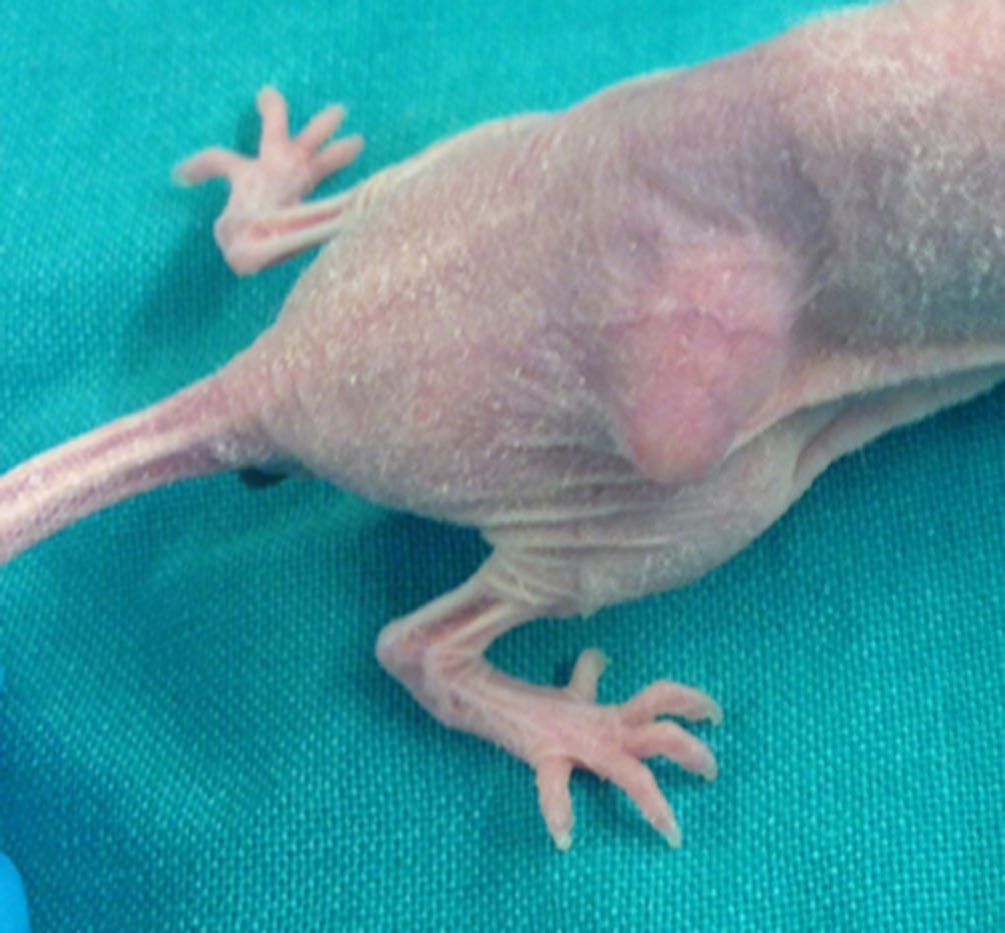
A mice exhibiting a subcutaneous heterotopic tumor—in this case, the cells were inoculated at the right side of dorsum

In the last decades, the xenograft model has been adopted as a way to bypass the limitations of the cell culture model. Tumor cell lines or suspensions are injected subcutaneously in mice, that in order to prevent tumor rejection usually are *nude* (unable to produce T cells) or have immune deficiency^34^—Figure 1. This type of model was a large success and nowadays it is widely used by researchers around the world to induce CRC in mice for carcinogenesis study and response to therapeutics.^42^, ^43^, ^44^ But like other models, the xenograft model has its limitations, one of them, the low metastatic capacity, reason that motivated some researchers to try new approaches like injection into the spleen, portal vein, and liver.^45^, ^46^, ^47^, ^48^, ^49^, ^50^, ^51^ The liver injection model does not represent a metastatic model but rather a heterotopic implantation of colon cancer cells and the spleen and portal vein injection models result in highly infiltrative tumors that impair the possibility of radiological characterization and treatment approaches.^5^

Other limitation of subcutaneous xenografts is the lack of reproduction of the tumor/microenvironment interaction—a well‐recognized element of predisposal to tumor indolent/aggressive behavior and distant metastases.^11^, ^15^, ^52^, ^53^, ^54^ The xenograft model allows the detection of cancer stem cells,^3^, ^55^, ^56^ but lacks the direct relation with local invasion and metastases. Despite its limitations, it is still amply used nowadays, especially in therapeutic studies.^57^

A way to overcome the lacunae in the previously described model was the development of a model where tumor cells are injected directly in the anatomical position of interest—thus giving birth to the orthotopic model, also in *nude* mice or with immune deficiency.^34^ Orthotopic models enhance the possibility of distant metastatic spread in a superior manner when compared with the subcutaneous models.^58^

In 1987 was created an orthotopic model of CRC in mice with injection of tumor cells in the ceacum, which enabled the study of local tumor invasion as well as metastatic dissemination—it was a more patient‐like animal tumor model.^59^ The success of this model was very high, turning it into a valuable asset in the study of the CRC, and amply used, with even some adaptations as injection of tumor cells in the rectum,^60^, ^61^, ^62^, ^63^, ^64^ and even exploring microvascular patterns of the colon concerning the differences between the mesenteric and antimesenteric side.^32^

**FIGURE 2:**
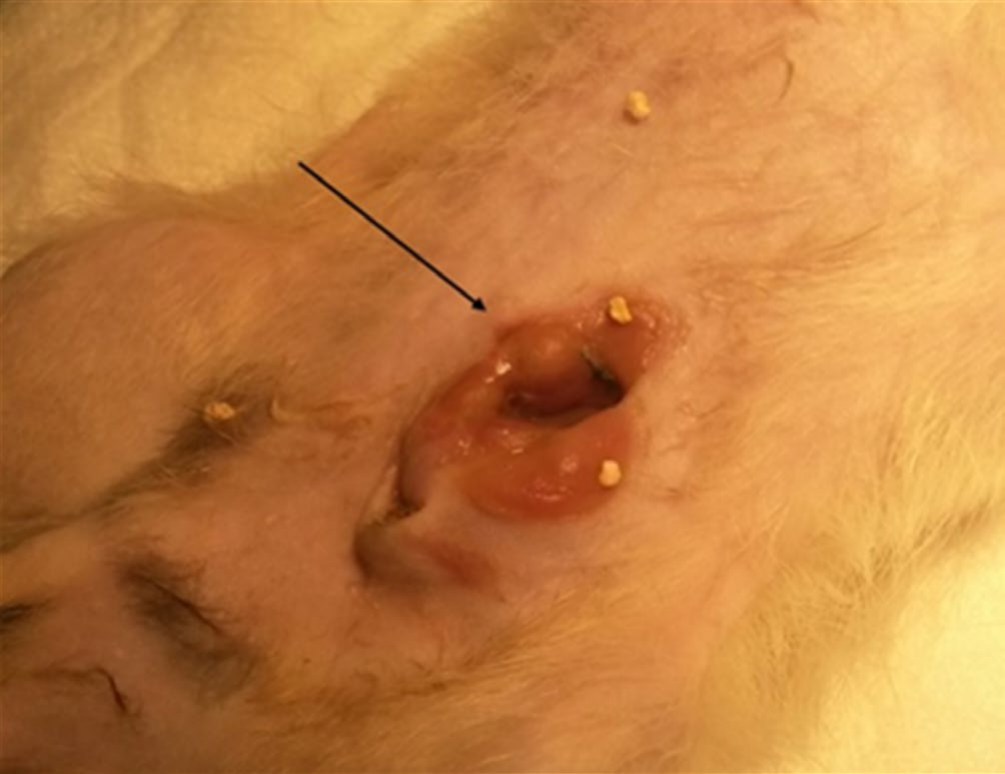
Colostomy with a nodular and submucosal lesion (black arrow), which on histological examination revealed an adenocarcinoma

The model was refined, with artificial selection of more aggressive CRC cells and the use of genetic engineering in order to create mice that were adequate to the studies.^65^ Finally, in 2009, with the improvement of surgical approaches and techniques, it was possible to create an orthotopic model recurring to a cecostomy surgical skill. This kind of model represented a major income/breakthrough due to the possibility of more sensitive tumor monitoring, real‐time visualization, and repeated tumor sampling^66^—Figure 2.

It must be referred, though, that the use of surgical skills (ostomy creations) for cancer studying had already been tried in 1994 with a double colostomy in the transverse colon and application of the chemical‐induced model principles.^67^

It seemed that the optimal mouse model for the study of CRC had been found. However, at the light of recent genomic studies of the colon and differences between proximal, transverse, distal, sigmoid, and rectosigmoid components,^68^, ^69^, ^70^, ^71^, ^72^ conjugated with the already known embryologic, anatomic, and physiological differences,^73^, ^74^, ^75^, ^76^ the high percentage of tumor in the left side of the colon^77^, ^78^, ^79^, ^80^ and with the knowledge that only orthotopic models for the right colon were described, it is easily perceived the lack of a “left side orthotopic tumor” model and its detailed study.

Bearing this in mind, in 2012, an orthotopic model for distal colon carcinoma was created, able to develop a distal colon cancer in vivo, that on a histological level induced tumors remarkably similar with human colon cancer. It resorted on the implantation of CRC cells in the submucosa of the distal colon of animals previously submitted to a descending colostomy with mucosal‐cutaneous fistula of the sigmoid colon, avoiding a fatal colon stenosis. However, it did not record the existence of metastatic disease.^81^ This model was further refined in 2016, with the use of different CRC cellular lines (different colonic origins—ascending, descending, rectosigmoid) in a murine model, which led to the development of distinct morphophysiological characteristics of the primary tumor with neural invasion and cancer stem cells identification, also similar to those observed in human disease; it is a simple and reproducible model of distal colon cancer, that enables the study of genetic and molecular pathways of CRC, their interaction with the microenvironment, and the study of the metastatic process.^82^

Some groups bypassed surgical skills and perform injections of colon cancer cells in the rectum and used mechanical means, like metallic stents, to overcome obstruction, with metastatic development; however, the results did not have statistical significance.^83^ The orthotopic model is a very interesting approach to CRC studies, since on a local level, it is very similar to what happens on human tissue, replicating human disease with high reliability; the need of surgical skills may be a lesser drawback, but many research on the literature resorts to this method.^84^, ^85^, ^86^

Still, the injection method has its limitations, with tumor developing rates that can go from 60% to 70%, which can be explained by incorrect parietal injection of tumor cells, low viability of tumor cells, and host reaction to the cells.^32^ Also, the metastases may not reach the metastatic site of interest, and when they do, sometimes they take a huge amount of time.^87^

In the last years, there has been application of different techniques to orthotopic models, to improve the tumor development success rate, such as electrocoagulation, with apparent maximization of tumor development, both locally and distant.^88^ Another approach was the development of patients' derived xenografts (PDX), which consist in the graft of tumor from human patients into an immune‐deficient animal, replicating in this manner the human scenario.^38^ Several approaches were made with PDX subcutaneous and orthotopic engraftments, reporting development of secondary disease in the orthotopic location, with nuclear medicine techniques and histological confirmation.^89^, ^90^, ^91^ The PDX models allow a strong preservation of the tumoral and stromal architecture, with a high degree of fidelity to the donor tumor—microscopic, genetic, and functional.^38^, ^90^

Despite all the favorable characteristics, the model presents some major limitations: samples are usually taken from patients with highly advanced tumors^92^ and from patients who had already undergone cycles of chemotherapy^90^; there is also concern about the amount of viable tumor being engrafted and intra‐tumoral heterogeneity^93^ and also the various strains of mouse and grafting techniques used.^90^ Recently, a new drawback has been raised with the demonstration that the human stroma is usually replaced by the murine stroma, regardless of the maintenance of histological characteristics of the tumor,^94^, ^95^ and it occurs very rapidly in CRC PDX.^96^

A similar approach has been performed, but using syngraft/isograft models, which consist of grafting tumor fragments or cells derived from 1 mouse into a genetically similar inbred and immune competent mouse.^97^ This method would allow bypassing the two major limitations of the xenograft models—species mismatch and the stroma issue, however the model is not human and it is highly time consuming and laborious.^98^

### 3.6 Genetic engineered mouse models

The development of gene targeting provided the possibility of genetic models of CRC, with many advantages because of the availability of genetic information and easy gene manipulation,^34^ especially in those relevant for the carcinogenic process.^58^

The evolution of genetic engineering and genetic manipulation techniques enabled the creation of models capable of replicating genetic abnormalities that cause CRC, such as: hereditary nonpolyposis CRC,^99^, ^100^, ^101^ familial adenomatous polyposis^102^, ^103^ and improvement of the APC heterozygous model^101^ and the modifier of Min model.^104^, ^105^

For most tumor types, the genetic engineered mouse models (GEMM) can be used to study the human disease, and even to develop therapeutics; however, the models only possess evidence in the early stages of disease, having little evidence in the advanced stages.^106^ GEMM models do not replicate the native process of metastasis, showing lower grade of dissemination and thus, needing more time to metastasize, and when metastases occur, they have a high grade of variability and are less reproducible,^107^ and mostly depend on concomitant Kras and Pr3 mutations.^108^ Besides, some GEMM of CRC (with *Apc* mutations) usually develops small bowel and not colon tumors, and tumor burden usually diminishes the lifespan of the animal limiting malignant progression with the majority of them not reporting secondary disease.^22^, ^98^

Additional limitations to the GEMM models include more heterogeneity in human tumors, which may be explained by a more varied diet and different microbiome in humans and consequently more exposition to toxins,^109^ different time of exposition to toxins (chronic exposition in humans and more time limited in mouse), lack of population genetic heterogeneity in mouse due to inbred ,and the fact that in GEMM tumor arise by the same genetic mutation which limits the number of genetic tumor pathways.^98^

Genetic engineered mouse models are particularly effective for the study of initial phases of disease but have not replaced the xenograft models as research tools for treatment methodologies in metastatic disease.^58^

The addition of new methodologies such as CRISPR‐Cas9 technology has provided plasticity to genomic editing and is appointed as a very successfully tool to achieve metastatic disease, especially when associated with CRC organoids;^108^ however, it is not a tool available in the majority of the laboratories.

## 4 CONCLUSION/FINAL CONSIDERATIONS

For the last years, many types of mouse models have been developed in order to study CRC. In the age of the genetic information and genetic manipulation techniques, it would seem that the GEMM models would be the most suitable for the task, but they have major limitations in the study of the later stages of the disease and in the metastatic process.

Of all the available models, the orthotopic model seems to have the “leading position” in cancer study because it allows the study of the tumoral microenvironment in vivo and the metastatic process. Because of the different characteristics of the colon segments, the creation of a model to study the carcinogenesis of the left colon is of major importance and presents a solution to the technical limitations of the other CRC models.

Each animal model has its pros and cons—Table 1, and in some cases, association of more than one form in CRC induction is necessary.^110^ The model should be chosen accordingly to the study expectations and aim, maximizing its potential.^111^

**TABLE 1:** Comparison between the different mouse models, with advantages and drawbacks

Animal model	Advantages	Drawbacks
Sporadic/chemical induced	✓ Easy to perform ✓ Similar to the carcinogenic process in human	✓ Low tumor development ✓ Wide variability in location, diffusion and differentiation ✓ Long time for tumor development
Carcinogens in situ	✓ Similar to the carcinogenic process in human ✓ Bypasses enzymatic alterations	✓ Low tumor development ✓ Long time for tumor development
Peritoneum models	✓ Easy to perform ✓ Quick results ✓ Good for antitumoral drugs tests	✓ Biological behavior does not mimic/replicate human tumors ✓ Biological behavior difficult to predict, usually with disseminated and advanced disease
Subcutaneous xenografts	✓ Use of human cancer cells ✓ Quick and easy to use	✓ Heterotopic inoculation of the tumor is not a physiologic process ✓ Low immune system activity ✓ Tumoral cancer cells and stromal cells are from different species ✓ Nonmetastatic ✓ Difficult to predict the response to antitumoral drugs
Orthotopic xenografts	✓ Use of human cancer cells ✓ Histology is similar to the human tumors ✓ Metastatic potential ✓ Replicates the local invasion process by the tumor, with lymphovascular invasion ✓ Allows genetic manipulation	✓ Low immune system activity ✓ Tumoral cancer cells and stromal cells are from different species ✓ Unable to replicate the initial steps of disease ✓ Less tumor formation than subcutaneous xenografts ✓ Difficult to predict the response to antitumoral drugs
Syngenic	✓ Tumor cells and stroma are from the same specie ✓ Intact immune system	✓ Endogenic animals does not allow the study of genetic modifiers ✓ Low number of metastases
Genetic engineered mouse models	✓ Genetic event is known ✓ In situ tumor development ✓ Reproduces early stages of oncogenesis ✓ Modified gene is expressed on physiologic level ✓ Tumor cells and stroma are from the same specie ✓ Intact immune system ✓ Used for chemoprevention studies	✓ Only partial replication of the human tumoral morphology and physiology ✓ Secondary mutations are different from the human tumors ✓ Rarely metastatic ✓ Response capacity to antitumoral drugs still unknown

Translational investigation for CRC is increasing, due to the high demand of proper models to study the complexity of in vivo biological behaviors. The application of the “left colon model” and its deep study will definitely bring new considerations about the carcinogenesis of rectosigmoid tumors, helping to unveil the pathways of metastization of CRC, the main cause of death in humans with the disease.
